# Draft Genome Sequence of Mobilitalea sibirica Strain P3M-3^T^, the Sole Representative of the Genus *Mobilitalea*

**DOI:** 10.1128/MRA.00129-21

**Published:** 2021-04-01

**Authors:** Nils Thieme, Regina Rettenmaier, Wolfgang Liebl, Vladimir V. Zverlov

**Affiliations:** a Chair of Microbiology, TUM School of Life Sciences Weihenstephan, Technical University of Munich, Freising, Germany; b Institute of Molecular Genetics, National Research Centre, Kurchatov Institute, Moscow, Russia; University of Southern California

## Abstract

Mobilitalea sibirica strain P3M-3^T^ is a strictly anaerobic, halotolerant, organotrophic bacterium of the family *Lachnospiraceae* that can utilize various plant-derived polysaccharides as its carbon source. The strain was originally isolated from a microbial mat in western Siberia (Russia). In this study, we present the draft genome sequence of *M. sibirica* P3M-3^T^ based on Illumina paired-end sequencing.

## Abstract (precis)

Mobilitalea sibirica strain P3M-3^T^ is a strictly anaerobic, halotolerant, organotrophic bacterium of the family *Lachnospiraceae* that can utilize various plant-derived polysaccharides as its carbon source. The strain was originally isolated by from a microbial mat in western Siberia (Russia). In this study, we present the draft genome sequence of *M. sibirica* P3M-3^T^ based on Illumina paired-end sequencing.

## ANNOUNCEMENT

The strain Mobilitalea sibirica P3M-3^T^ (phylum *Firmicutes*, order *Clostridiales*, family *Lachnospiraceae*) is a mesophilic and strictly anaerobic bacterium that was isolated in western Siberia (Russia) from a microbial mat (1). P3M-3^T^ utilizes starch, xylan, and crystalline cellulose as well as other polysaccharides as its carbon source. The main products of its fermentation are acetate, ethanol, H_2_, and CO_2_. The ability to depolymerize a wide array of polysaccharides makes *M. sibirica* an intriguing candidate for biorefinery approaches, e.g., microbial cocultures (2). Since strain P3M-3^T^ is the first and sole representative of the genus *Mobilitalea*, its genome sequence information is essential for targeted genetic engineering. Therefore, we created a draft genome sequence of *M. sibirica* P3M-3^T^ in this study using Illumina paired-end sequencing.

*M. sibirica* P3M-3^T^ (=DSM 26468^T^) was obtained from the German Collection of Microorganisms and Cell Cultures (DSMZ). The strain was cultivated anaerobically in GS2 medium (3) with 0.5% (wt/vol) cellobiose (Merck, Darmstadt, Germany) as the carbon source at 37°C. High-molecular-weight (HMW) DNA was extracted from a culture using the MagAttract HMW DNA kit from Qiagen (Hilden, Germany). The 16S rRNA gene was amplified and later sequenced with the primers 27F (AGAGTTTGATCMTGGCTCAG) and 1492R (CGGTTACCTTGTTACGACTT). The clonal purity of the strain was confirmed through Sanger sequencing by GeneWiz (South Plainfield, NJ, USA). At least 1 μg of chromosomal DNA was used to prepare a DNA library with the TruSeq DNA PCR-free sample preparation kit (Illumina, San Diego, CA, USA) (4). The library was sequenced using the Illumina MiSeq system in paired-end mode, according to the manufacturer’s instructions. The read length was 150 bp, and 4,859,739 forward reads as well as 4,859,739 reverse reads were generated. Read assembly was performed using SPAdes v3.13.0 (5), and default parameters were used. The BayesHammer package (6) is bundled in SPAdes v3.13.0, and it was used with default settings for read error correction. A total of 40 contigs were assembled with a 40-fold coverage and an *N*_50_ value of 165,485 bp. The combined length of the contigs is 3,854,872 bp, with a G+C content of 36.55 mol%. Open reading frames (ORFs) were predicted and annotated using the NCBI Prokaryotic Genome Annotation Pipeline (PGAP) (7), resulting in 3,332 coding sequences and 74 RNAs (3 5S rRNAs, 7 16S rRNAs, 7 23S rRNAs, 53 tRNAs, and 4 noncoding RNAs [ncRNAs]).

The genome sequence presented in this work is a valuable resource for evaluating *M. sibirica* P3M-3^T^ for future biotechnological applications. Furthermore, the genome sequence also allows us to gather more insights into the genetic properties and phylogeny of the genus *Mobilitalea*.

### Data availability.

This whole-genome shotgun project has been deposited at DDBJ/ENA/GenBank under the accession number JAEAGR000000000. The version described in this paper is version JAEAGR000000000.1. The raw sequencing reads are provided in the Sequence Read Archive (SRA) under the accession number SRR13181447. The whole project is summarized under the BioProject accession number PRJNA682100 and contains one BioSample under the accession number SAMN16980878.
